# Hypercalcemia and Flower Cells on Peripheral Smear Revealing Human T-lymphotropic Virus Type 1 (HTLV-1)-Associated Adult T-cell Leukemia/Lymphoma

**DOI:** 10.7759/cureus.103225

**Published:** 2026-02-08

**Authors:** Jeremy M Williams, Barrie Clark, Jorge Verdecia, Julia An

**Affiliations:** 1 Internal Medicine, University of Florida College of Medicine – Jacksonville, Jacksonville, USA; 2 Infectious Diseases, University of Florida College of Medicine – Jacksonville, Jacksonville, USA; 3 Pathology, University of Florida College of Medicine – Jacksonville, Jacksonville, USA

**Keywords:** adult t‑cell leukemia/lymphoma (atll), flower cells, human t‑cell leukemia virus type 1 (htlv‑1), hypercalcemia/hypercalcemic crisis, non‑endemic region/low‑prevalence setting, peripheral blood smear, t‑cell neoplasm/t‑cell lymphoma

## Abstract

Adult T-cell leukemia/lymphoma (ATLL) is a rare, aggressive peripheral T-cell malignancy caused by human T-lymphotropic virus type 1 (HTLV-1), most commonly affecting individuals from endemic regions. Diagnosis is challenging and often delayed due to low clinical suspicion and overlapping features with other hematologic and systemic disorders. A characteristic finding on peripheral blood smear is the presence of “flower cells,” atypical lymphocytes with deeply lobulated, petal-like, or cloverleaf nuclei and condensed chromatin, which is considered highly suggestive of acute-type disease. This case describes a 59-year-old female diagnosed with ATLL presenting with severe hypercalcemia and characteristic flower cells on peripheral smear, despite lacking recognized epidemiologic risk factors for HTLV-1 infection.

## Introduction

Human T-lymphotropic virus type 1 (HTLV-1) is an enveloped, single-stranded RNA retrovirus that causes several inflammatory and immunologic diseases and rarely progresses to adult T-cell leukemia/lymphoma (ATLL) or tropical spastic paresis/HTLV-1-associated myelopathy [1-4]. It spreads via cell-to-cell contact, enabling rapid dissemination, evasion of the host immune response, and low viremia [1]. Transmission occurs via blood and body fluids, including mother‑to‑child (especially breastfeeding), sexual intercourse, and parenteral exposure [1,2]. The virus preferentially infects CD4+ CCR4+ T-cells and establishes lifelong infection through proviral integration and subsequent clonal expansion of infected cells, a process that underlies its long latency and oncogenic potential [1-3]. Acute infections are often asymptomatic and go undetected, thereby allowing the virus to replicate for decades before noticeable symptoms appear; only 2-5% of HTLV-1 carriers progress to ATLL [2,5].

​Among the malignant sequelae of chronic HTLV‑1 infection, ATLL is a highly aggressive peripheral T‑cell neoplasm and is classified into four clinical subtypes: acute, lymphomatous, chronic, and smoldering [1]. These subtypes are differentiated by peripheral blood involvement, symptom severity, organ infiltration, and biochemical abnormalities [6]. While chronic and smoldering subtypes are more favorable and indolent in nature, the acute and lymphomatous forms are more aggressive and typically present with generalized lymphadenopathy, skin lesions, hepatosplenomegaly, and often severe hypercalcemia [7]. Hypercalcemia, if present, is a poor prognostic indicator and is mediated by osteoclast activation via parathyroid hormone-related peptide (PTHrP), receptor activator of nuclear factor κB ligand (RANKL), and proinflammatory cytokines produced by malignant T-cells [6,7]. The diagnosis of ATLL relies on integration of clinical features with laboratory evidence of a mature peripheral T‑cell neoplasm and concomitant HTLV‑1 seropositivity. Peripheral blood smear may reveal “flower cells,” atypical lymphocytes with deeply lobulated, petal‑like nuclei and condensed chromatin, which are highly characteristic of acute‑type ATLL and can provide an early morphologic clue to the diagnosis [8].

HTLV-1 is endemic in regions including Japan, Brazil, the Middle East, the Caribbean, and Africa [2,5]. The prevalence of HTLV-1 in the general U.S. population is low, with approximately 5.1 cases per 100,000 individuals. However, prevalence is notably higher in communities with larger proportions of immigrants from endemic regions or higher rates of intravenous drug use [5]. In the United States, the incidence of ATLL is estimated at 0.05 cases per 100,000, with most cases occurring among individuals from endemic regions [9]. Consequently, outside endemic areas, diagnosis of HTLV-1/ATLL is often delayed due to low clinical suspicion and presentations that overlap with those of other lymphomas or infections, particularly when hypercalcemia is the initial or predominant manifestation, given the presence of several more common etiologies.

This report describes a case of HTLV‑1-associated ATLL presenting with life‑threatening hypercalcemia in a patient living in the United States without obvious epidemiologic risk factors. This case highlights the unique diagnostic and therapeutic challenges that clinicians face in non‑endemic settings.

## Case presentation

A 59-year-old female with a history of hypertension, chronic kidney disease, HIV on antiretroviral therapy, and vitamin D deficiency presented with complaints of worsening muscle weakness, confusion, and multiple falls over the past two days. She endorsed three mechanical falls during this time, which were precipitated by her “legs giving out.” The last fall occurred just prior to arrival, as she struck the back of her head on the counter but did not lose consciousness. She denied any recent chest pain, palpitations, fevers, vision changes, numbness, or paresthesias. Home medications include abacavir-dolutegravir-lamivudine 600-50-300 mg daily, amlodipine-benazepril 5-20 mg daily, and cholecalciferol (D3) 2000 IU daily. She endorsed compliance with all medications and denies any medication changes, alcohol, or illicit drug use prior to symptom onset.​

On arrival, vital signs were within normal limits; however, the patient was alert and oriented to self only. The physical examination was significant for obesity, a small superficial contusion on the posterior scalp, and 2/5 muscle weakness in bilateral upper and lower extremities without evidence of fasciculations, atrophy, or hypertonicity. Laboratory investigation (Table 1) was notable for hypercalcemia (calcium 18.8 mg/dL), hyperkalemia (potassium 5.6 mmol/L), and acute kidney injury (creatinine 3.37 mg/dL; baseline 1.4 mg/dL). Further workup revealed a normal complete blood count, normal parathyroid hormone and parathyroid hormone-related protein levels, and normal creatine kinase, thyroid-stimulating hormone, 1,25-dihydroxyvitamin D, and 25-hydroxyvitamin D. Imaging showed diffuse mediastinal and axillary lymphadenopathy. Still, he was negative for acute fracture/dislocation, intracranial abnormalities, or discrete masses.

**Table 1:** Patient's laboratory investigations at presentation with reference ranges for comparison

Laboratory investigation	Patient value	Reference range
Calcium	18.8 mg/dL	8.6-10.0 mg/dL
Potassium	5.6 mmol/L	3.4-4.5 mmol/L
Creatinine	3.37 mg/dL	0.51-0.95 mg/dL
Parathyroid hormone	27 pg/mL	16-65 pg/mL
Parathyroid hormone-related protein	<2.0 pmol/L	<2.0 pmol/L
Creatinine kinase	27 U/L	22-195 U/L
Thyroid-stimulating hormone	1.65 mIU/L	0.270-4.200 mIU/L
1-25-Dihydroxy vitamin D	13.0 pg/mL	24.8-81.5 pg/mL
25-Hydroxy vitamin D	34.4 ng/mL	30.0-100.0 ng/mL

She was started on continuous IV fluids with normal saline (300 cc/hr) and calcitonin (4 units/kg Q12 hours) for treatment of hypercalcemia. After 48 hours, the patient's hypercalcemia and renal function showed minimal improvement and were subsequently deemed refractory. Nephrology was consulted, and the patient underwent two sessions of intermittent hemodialysis, with rapid correction of calcium levels and renal function, and mentation returned to alert and oriented x3.

**Figure 1:**
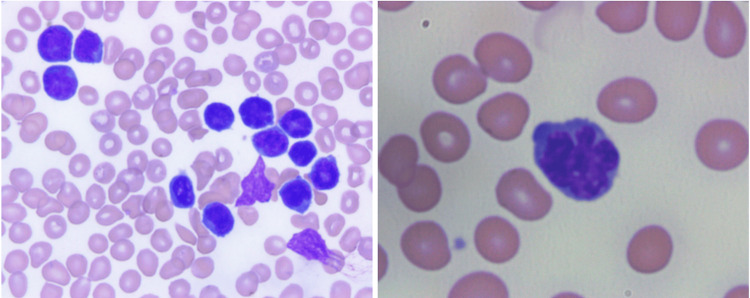
Peripheral blood smear showing "flower cell," an atypical lymphocyte with deeply lobulated, petal-like, or cloverleaf nuclei and condensed chromatin, consistent with ATLL acute subtype ATLL: adult T-cell leukemia/lymphoma

​After the patient was clinically stabilized, further investigation into the etiology of the patient's hypercalcemia was pursued, particularly a malignancy workup, given the grossly unremarkable findings up to that point in the hospitalization. On chart review, the patient was noted to be up-to-date and negative for all age-appropriate cancer screenings, including breast, cervical, and colon. A complete osseous skeletal survey was performed and was negative for lytic or blastic lesions. Both serum and urine protein electrophoresis were negative for evidence of monoclonal gammopathy. Flow cytometry of peripheral blood showed an immunophenotypically abnormal CD4(+)/CD7(-) T-cell population of 9.3%, no blasts, and otherwise normal mature immunophenotypes of both monocyte and granulocyte populations. However, morphologic review of the corresponding peripheral blood smear identified atypical lymphocytes with a high nuclear-to-cytoplasmic ratio, mildly condensed chromatin, nucleoli, and "flower"-like nuclei, comprising approximately 10% of all nucleated cells (Figure 1).

The abnormal findings in Figure 1 prompted a qualitative serologic assessment for HTLV-1 antibodies, which was positive. These findings, in combination with the flow cytometric immunophenotypic analysis, supported a diagnosis of ATLL. Subsequent immunophenotypic testing, supported by excisional lymph node and bone marrow biopsies, confirmed the diagnosis. Hematology/oncology was consulted, and the patient was started on chemotherapy with cyclophosphamide, hydroxydaunorubicin, oncovin, and prednisone (CHOP). The patient showed clinical improvement with resolution of her hypercalcemia, renal function, and altered mentation. She was discharged from the hospital in stable condition with a plan for outpatient follow-up with oncology for continuation of chemotherapy.

## Discussion

This case highlights severe, malignant hypercalcemia as the initial manifestation of HTLV-1-associated ATLL in a patient with HIV and without clear epidemiologic exposure to an endemic HTLV‑1 region. Hypercalcemia is common and often profound in aggressive ATLL, but it more typically appears in the setting of overt leukemia/lymphoma or in patients with known or suspected HTLV‑1 infection [10]. ATLL frequently presents with lymphadenopathy, skin involvement, circulating “flower cells,” and hypercalcemia [2,4]. Hypercalcemia has been reported in roughly 50-70% of ATLL cases and is a strong adverse prognostic marker [11]. Several case reports and series describe hypercalcemia as the first clue to ATLL, but many involve patients from highly endemic regions or with obvious epidemiologic risk factors. In contrast, this patient resided in the United States, had negative age‑appropriate cancer screening, and carried a longstanding HIV diagnosis without prior recognition of HTLV‑1 co‑infection, paralleling only a small number of reported North American and co‑infected cases [11-13].

​Hypercalcemia in ATLL is primarily mediated by osteoclast activation via RANKL overexpression, PTHrP, and proinflammatory cytokines produced by malignant T-cells, with bone marrow infiltration and lytic lesions present to varying degrees [11]. The current case underscores that, even in the absence of radiographic bone disease and despite low PTHrP levels, paraneoplastic osteoclast activation can produce marked elevations in calcium. Chronic HTLV‑1 infection, clonal expansion of infected CD4+ T-cells, and the actions of viral proteins are thought to contribute to pathogenesis [1,3,4]. This case also illustrates the difficulties of managing malignant hypercalcemia, specifically in the setting of undiagnosed ATLL, as it often manifests as severe, recurrent, and treatment‑resistant.

​Diagnosing ATLL outside endemic regions is challenging because its symptoms are vague and heterogeneous, overlapping with those of many other pathologies. Studies of Western ATLL show that many patients present with advanced, aggressive disease; hypercalcemia and widespread nodal involvement are frequent, under-recognized clues [10-12]. In this case, flow cytometry and peripheral blood smear revealed atypical CD4+/CD7- T-cells with “flower-like” nuclei. Lymph node and bone marrow biopsies confirmed the diagnosis, highlighting the importance of combining morphology, immunophenotyping, and HTLV-1 serology.

​HTLV-1-associated ATLL has a generally poor prognosis, with median survival often limited to months despite multi-agent chemotherapy [13]. Hypercalcemia consistently predicts worse outcomes. Current data support risk-adapted use of combination chemotherapy, antiretroviral-interferon-based regimens, allogeneic stem cell transplantation when feasible, and exploration of targeted and immune-based therapies [7,12,14]. In this patient, CHOP-based therapy and supportive care resulted in rapid, clinically meaningful improvement in calcium levels and mental status. However, long-term outcomes remain guarded.

​This case expands the known presentations of HTLV‑1/ATLL in non‑endemic regions. Malignant hypercalcemia, especially if a secondary workup is unremarkable, should prompt early consideration of HTLV‑1/ATLL, even if PTHrP and vitamin D results are inconclusive. Early hematologic evaluation is critical. A careful review of the peripheral smear and targeted flow cytometry provides key diagnostic information in patients with suspected malignant hypercalcemia or unexplained lymphadenopathy. This approach supports early detection of T-cell neoplasms, guided HTLV‑1 testing, and quicker initiation of treatment to improve patient outcomes.

## Conclusions

This case illustrates life‑threatening hypercalcemia as the initial manifestation of HTLV‑1-associated adult T‑cell leukemia/lymphoma in a patient without recognized epidemiologic risk factors, underscoring the diagnostic challenges in non‑endemic settings. The evaluation began with the identification of circulating “flower cells” using peripheral smear review. This finding prompted immunophenotyping, which revealed an abnormal CD4+/CD7- T‑cell population, followed by confirmatory HTLV‑1 serology and tissue biopsies. This diagnostic sequence underscores the importance of integrating peripheral smear review, immunophenotyping, and targeted virologic testing when evaluating unexplained malignant hypercalcemia. Clinicians should maintain a high index of suspicion for ATLL in patients with severe or refractory hypercalcemia and atypical lymphocytosis, even when PTHrP and vitamin D studies are non‑diagnostic and traditional risk factors for HTLV‑1 infection are not apparent, as timely recognition can facilitate prompt initiation of therapy and potentially improve outcomes.
